# Assessment of utility values and QALYs after primary PCI with DP-Xience and BP-Biomatrix stents

**DOI:** 10.1371/journal.pone.0253290

**Published:** 2021-06-17

**Authors:** Salma Bibi, Amjad Khan, Asif Nadeem, Saima Mushtaq, Gul Majid Khan

**Affiliations:** 1 Department of Pharmacy, Quaid-i-Azam University, Islamabad, Pakistan; 2 Armed Forces Institute of Cardiology-National Institute of Heart Diseases (AFIC-NIHD), Rawalpindi, Pakistan; 3 Department of Healthcare Biotechnology, Atta-ur-Rahman School of Applied Biosciences, National University of Sciences and Technology, Islamabad, Pakistan; 4 Islamia College University, Peshawar, Khyber Pakhtunkhwa, Pakistan; Government College University Faisalabad, PAKISTAN

## Abstract

**Background:**

Primary percutaneous coronary intervention (PPCI) is the recommended treatment in ST elevated myocardial infarction (STEMI). The determination of Quality of life (QoL) for various options of coronary revascularization is important for establishment of a comprehensive care plan. Studies of QoL in interventional cardiology are scarce. Our study has compared utility scores and quality adjusted life year (QALY) of 2^nd^ and 3^rd^ generation drug eluting stents (DES).

**Methods:**

An observational cohort study was conducted to evaluate QoL and QALY using EQ-5D-5L questionnaire. Patients undergoing PPCI between July-Dec 2019 were evaluated after completion of one year of procedure.

**Results:**

Total 334 patients were evaluated, study population consisted of a greater number of males (87.13%) than females. Mean utility value was more in 3^rd^ G Biomatrix stents; 0.829 ± 0.11 than 2^nd^ G Xience stents; 0.794 ± 0.11 (p < 0.05). Visual analogue scale (VAS) value was also high in 3^rd^ G DES (81.84 ± 8.29) as compared to 2^nd^ G DES (77.81 ± 9.01); p< 0.05. A significant association was found between utility scores/VAS and age, DM, HTN, Current smoking, family history and CAD diagnosis. There was a gain of 0.035 QALY with the use of Biomatrix DES.

**Conclusion:**

Health related quality of life (HRQOL) is a leading support in the decision making of therapeutic interventions. Our study has found that Biodegradable polymer (BP) Biomatrix DES are superior to the Durable polymer (DP) Xience DES having better QoL and QALY.

## 1. Background

Worldwide, the second major cause of death has been the coronary artery disease (CAD) [1, 2]. It increases the rate of mortality as well as affects the HRQOL badly, and has a negative impact on the vitality, energy levels, psychosocial aspects and social interaction. CAD patients show a significant reduction in the levels of morbidity and mortality after percutaneous coronary intervention (PCI) [3, 4]. Health as defined by the World Health Organization (WHO 1947) is “not merely the absence of disease but also the physical, mental and social welfare”. This definition depicts that the assessment of health not merely includes the length of life and the absence/presence of disease but also considers the “quality of life” [5].

WHO defines the quality of life as the ‘individuals’ perception of their position in life in context of their culture and value systems in which they live, and in relation of standards, goals, concerns and expectations [6]. Improvement in the QoL of patients is the most usual indication for PCI in CAD patients after STEMI [7, 8]. By assessing the QoL and its determinants, clinical practice and research gaps can be reduced [9]. CAD poses a huge burden on communities in the world due to adverse effects on QoL, pre-mature mortality and economic status of families. Therefore, the goal of CAD therapies should not be only to increase the life span but also the improvement in QoL. Patients expect functional recovery who receive treatments like PCI and coronary artery bypass graft (CABG) for relieving their symptoms. Quality of life after CAD is affected by gender, age, education, marital status, obesity, treatment adherence and co-morbidities [4, 10]. Dyslipidemia has been found to have association with the development of poor HRQOL after PCI [11]. Cigarette smoking has also been identified as a risk factor of CAD and is involved in possession of poor QoL [12].

Quality adjusted life year (QALY) is defined as a standardized measure of the disease burden that combines the health-related quality of life and survival as one index. It is basically used for the cost effectiveness evaluation studies to guide the decision makers for allocation of limited healthcare resources [13]. Due to the property of comparing impact of different therapies at once, QALY is often recommended and used as a health outcomes measure [6]. There are many methods to date to assess the QoL in cardiovascular disease patients, which include EQ-5D-5L, Seattle Angina Questionnaire and MacNew Heart Disease, etc. [5, 10, 14]. EQ-5D-5L is the preferred instrument for evaluation of QoL by the National Institute of Health and Care Excellence (NICE) UK, the Canadian agency for drugs and technology in health (CADTH), the European network of technology assessment (EunetHTA) and other decision making bodies [15, 16]. Utility values obtained by EQ-5D-5L questionnaire can be used for assessment of QALYs [3].

The industry of coronary stents is advancing at a very fast pace. Durable polymer drug eluting stents (DP-DES) have many disadvantages including the presence of permanent polymer after completion of drug release process, biodegradable polymer (BP) stents overcome these issues and leave a polymer free stents after releasing anti-proliferative drug [17]. Studies on PCI mainly focus on the outcomes and very few studies are available on quality of life. The recognition of measuring quality of life for assessment of treatment outcomes of chronic illness has been increased recently among researchers and clinicians. HRQOL is also used in calculations of cost effectiveness analysis of different types of therapeutic options. The determination of QoL of various options for coronary revascularization is important for establishment of a comprehensive care plan. PPCI is the first line treatment for STEMI [18, 19]. Studies on post-PCI and post-PPCI QoL are very limited [20]. Therefore, this study was aimed to compare the quality of life, quality adjusted life years and predictors of QoL in coronary artery disease patients undergoing PPCI with 2^nd^ and 3^rd^ generation drug eluting stents.

## 2. Methodology

### Study design

An observational cohort study was conducted to evaluate the QoL and quality adjusted life years in CAD patients who have undergone primary percutaneous coronary intervention with 2^nd^ G (Abbott’s Everolimus eluting Xience Prime & Xience Xpedition) or 3^rd^ G (Biosensors’ Bolimus eluting Biomatrix Neoflex & Biomatrix Alpha) DES on cardiologist discretion. Xience Xpedition Everolimus eluting stent (EES) by Abbott has a cobalt chromium strut, a durable, non-erodible polymer loaded with 100 ug/cm^2^ everolimus [21]. Xience prime (Abbott) contains a balloon expandable delivery system and Everolimus (100 ug/cm^2^) coated stent [22]. BioMatrix Biolimus eluting stent (BES) manufactured by Biosensors (Switzerland) are stainless steel platform stents having polylactic acid (PLA) as biodegradable polymer [23]. BP-BES (Biomatrix Neoflex) is indicated for improvement of luminal diameter of coronary arteries. BES was the first biodegradable polymer DES. BioMatrix alpha (Biosciences International) is made of cobalt-chromium thin strut [24].

### Study cohort

**Fig 1:**
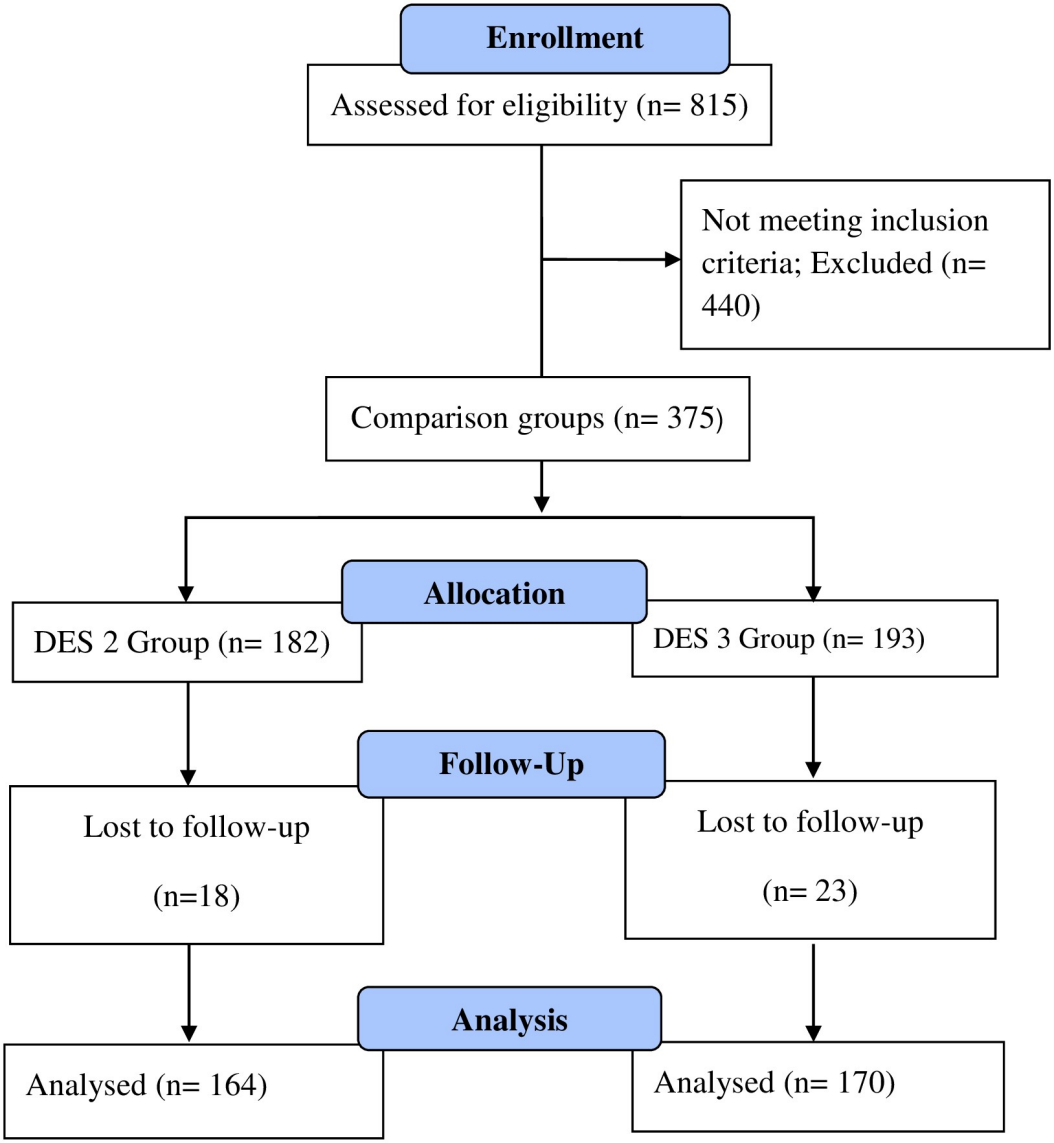
CONSORT flow diagram.

The current study was approved by Bioethics committee of Quaid-i-Azam University Islamabad (vide letter no. BEC-FBS-QAU2020-243) & Institutional and Ethical review board (IERB) of Armed forces Institute of Cardiology- National Institute of heart diseases (AFIC- NIHD), Rawalpindi, Pakistan (letter No. 10/11/R&D/2020/95). This study has been conducted in outdoor patient department of AFIC-NIHD. Sample size was not calculated and all the patients meeting inclusion/exclusion criteria were included in study. Patients who have visited the study center between Jul-Dec 2019 and received a single Xience Prime/Xience Xpedition or Biomatrix Alpha/Biomatrix neoflex stent on cardiologist discretion were recruited in study. Patients less than 18 years of age; undergoing PPCI other than CAD; receiving bare metal stents (BMS), 1^st^ G DES, or 2^nd^/3^rd^ G other than mentioned above; patients having previous history of CABG, Plain Old Balloon Angioplasty (POBA) or PCI; patients receiving more than one stent were excluded. In total, 815 patients were enrolled, patients not fulfilling the inclusion criteria were dropped, 34 patients lost to follow-up and 334 patients completed follow-up (Fig 1).

### Data collection

Patient’s demographic details, reason for PCI, stent type and angiographic characteristics were retrieved from the hospital’s individual patient record. Patients were interviewed at follow-up visits after completion of one year from the date of PPCI. Past medical history and risk factor assessment were done. The data collection form was designed for obtaining demographic details and history of patients. To estimate comparative improvement in patient’s quality of life brought by 2^nd^ (xience prime/xience xpedition) or 3^rd^ (biomatrix neoflex, biomatrix alpha) generation stents, EQ-5D-5L questionnaire was administered at completion of one year of procedure.

### Study tool

EQ-5D-5L is a validated questionnaire for determining QoL from EuroQol. This study was registered at EuroQol site under registration ID: 36945 and patient’s self-administered paper version questionnaire (Urdu) was obtained, telephonic version was used for patients who had not visited hospital for follow-up. EQ-5D-5L contains five areas: mobility, self-care, usual activities, pain/discomfort and anxiety/depression. The questionnaire consisted of two parts; EQ-5D-5L contains five dimensions and patients marked their health status by ticking a box opposite to statement against each dimension. Response of EQ-5D-5L was obtained in the form of a 5-digit number that indicates health profile of individual patients. The response of questionnaire has a total 3125 possibilities. Health state index score was then calculated based upon UK based value sets (most commonly used) as Pakistan based value sets were not available, index score is also called as ‘utilities’. EQ-VAS is a scale that has value from 0–100, “0” indicates worst health and “100” indicates best health. Patients marked their health status on VAS, this gave the qualitative measure of their health, VAS measures quality from the patient’s perspective.

### Statistical analysis

Statistical software package for social sciences (IBM SPSS statistics version 21) has been used for evaluation of data. Categorical variables were presented as frequencies and percentages and a chi-square test was used for obtaining their p-values. Numerical variables were presented as mean and standard deviation and the Man Whitney test was used for their comparison. Multiple Linear Regression/Multiple Regression Analysis was used to obtain association of utility scores and visual analogue scale values with demographic variables, angiographic variables and risk factors. Results were presented as unstandardized co-efficient B, 95% confidence interval and P-value. A P-value less than 0.05 was considered as statistically significant.

## 3. Results

### Demographic/angiographic characteristics and risk factors

Demographic and angiographic characteristics of study population (gender, age, residence, occupation status, type of MI, CAD diagnosis) are presented in Table 1. Study population consisted of a greater number of males (87.13%) than females (12.87%) and 4 age groups were classified, age group 58–75 years included the highest number of patients (52.3%). Two groups of study population were created on the basis of their residential status, i.e., urban (70.1%) and rural (29.1%). Anterior MI pattern appeared in 67.45% (n = 230) patients. In study population, radial access was used for all patients. Stent’s length ranged from 14-74mm (mean stent length **28.86 ± 8.679**). Mean stent length in 2^nd^ generation DES was 29.39mm ± 9.825 and for 3^rd^ generation DES 28.86mm ± 7.451. The study population consisted of 20.3% diabetic, 32.9% hypertensive, 18.9% having hyperlipidemia, 17.66% current smokers and 28.44% having a positive family history of CAD.

**Table 1:** Distribution of utility values with demographic, angiographic variables and risk factors.

Sr. No	Variable	Categories	Frequency (334)	Mean Utility Value (SD)
1	**Gender**	Male	291 (87.13%)	0.8218 (0.11)
		Female	43 (12.87%)	0.773 (0.12)
2	**Age**	19–38	7 (2.1%)	0.834 (0.12)
		39–57	143 (42.8%)	0.834 (0.11)
		58–75	175 (52.3%)	0.794 (0.11)
		> 75	9 (2.7%)	0.793 (0.12)
3	**Residence**	Urban	234 (70.1%)	0.809 (0.11)
		Rural	100 (29.9%)	0.821 (0.10)
4	**Occupation**	Unemployed	160	0.812 (0.11)
		Employed	174	0.812 (0.11)
5	**DM**	Yes	68 (20.3%)	0.744 (0.10)
		No	266 (79.64%)	0.829 (0.11)
6	**HTN**	Yes	110 (32.9%)	0.746 (0.10)
		No	224 (67.07%)	0.844 (0.10)
7	**Hyperlipidemia**	Yes	66 (19.8%)	0.769 (0.09)
		No	268 (80.24%)	0.823 (0.11)
8	**Current Smoking**	Yes	59 (17.66%)	0.751 (0.10)
		No	275 (82.33%)	0.825 (0.11)
9	**Family History**	Yes	95 (28.44%)	0.783 (0.11)
		No	239 (71.56%)	0.824 (0.11)
10	**Type of MI**	ANT MI	226 (67.66%)	0.816 (0.11)
		INF MI	108 (32.34%)	0.804 (0.10)
11	**CAD Diagnosis**	SVCAD	129 (38.62%)	0.836 (0.11)
		DVCAD	108 (32.34%)	0.811 (0.12)
		TVCAD	96 (28.74%)	0.780 (0.10)
12	**DES Type**	2^nd^ G	164 (49.1%)	0.794 (0.11)
		3^rd^ G	170 (50.9%)	0.829 (0.11)

Abbreviation: DM: Diabetes mellitus; HTN: Hypertension; MI: Myocardial Infarction; DES: Drug eluting stents.

### EQ-5D-5L utility score

Values of questionnaire EQ-5D-5L were obtained in the form of a five digit number. A health state index score (utility value) was calculated for each health state value as per UK based value set. 16 health states were observed in the study population. Complete healthy state (11111; Utility score: 1.00) was observed in twenty-six (7.8%) respondents, worst health state in our study population was 33321, observed in 13 patients (utility score: 0.61) (Table 2).

**Table 2:** Health states of PPCI patients.

Sr. #	EQ-5D Health states	Frequency	Index value*
1	11111	26	1.00
2	11121	27	0.94
3	12111	18	0.911
4	12121	7	0.851
5	12211	11	0.843
6	12221	13	0.783
7	21111	27	0.904
8	21121	73	0.844
9	21211	7	0.836
10	21221	20	0.776
11	22111	3	0.815
12	22121	15	0.755
13	22221	49	0.687
14	22222	4	0.63
15	22231	21	0.649
16	33321	13	0.61
**Total**		**334**	

*Index values were obtained by UK based value sets.

Utility is a value in between 0–1, 1 indicates perfect health and ‘0’ indicates death. Mean utility value was high in 3^rd^ G DES, i.e., 0.829 ± 0.11 than 2^nd^ Generation DES group 0.794 ± 0.11. Utility value was more in males (0.8218 ± 0.11 vs 0.773 ± 0.12) and age group 39–57 (0.834 ± 0.11). In patients of urban and rural areas, value of utility was almost same (0.809 ± 0.11 & 0.821 ± 0.10 Resp.). Utility scores were less for patients having diabetes mellitus (0.744 ± 0.10), hypertension (0.746 ± 0.10), hyperlipidemia (0.769 ± 0.09), current smoking status (0.751 ± 0.10) and family history of CAD (0.783 ± 0.11) (Table 1).

A multiple regression/multiple linear regression analysis was run to predict the utility values from age, gender, DM, HTN, Current smoking, family history of CAD, hyperlipidemia, DES type, Type of MI and CAD diagnosis. These variables statistically significantly predicted the utility score, F (10, 322) = 20.257, p < 0.0005, R^2^ = 0.386, Adjusted R^2^ = 0.367. A significant association was found between utility values and DES type (p< 0.05). Except 2 variables (hyperlipidemia and type of MI), all others (age, gender, DM, HTN, smoking, family history of CAD and CAD diagnosis) were found to have a statistically significant relation with response variable (Utility value) i.e., P < 0.05 (Table 3).

**Table 3:** Factors associated with utility scores (EQ-5D-5L).

Sr. No.	Variable	Unstandardized Co-efficient (B)	Std. Error	p-value (Sig.)	95% CI for B
**1**	**Age**	-.023	.008	.006	-0.040 - -.0.007
**2**	**Gender**	-.031	.015	.042	-0.061–0.001
**3**	**DM**	.059	.013	.000	0.035–0.084
**4**	**Hypertension**	.069	.011	.000	0.047–0.090
**5**	**Current Smokers**	.081	.013	.000	0.056–0.107
**6**	**Family History**	.032	.011	.004	0.010–0.054
**7**	**Hyperlipidemia**	.023	.013	.065	-0.001–0.048
**8**	**DES Type**	-.035	.010	.000	-0.054 - -0.016
**9**	**Type of MI**	-.009	.010	.390	-0.029–0.012
**10**	**CAD Diagnosis**	-.017	.006	.006	-0.029 - -0.005

### Quality adjusted life years

QALY is used to quantify the effectiveness of therapy. It is calculated by using a formula “years of life * utility” (QALY = Years of life * Utility). QALY value for 2^nd^ G DP-DES was 0.794 (QALY = 1 * 0.794 = 0.794 QALY) and for 3^rd^ G BP-DES 0.829 (QALY = 1 * 0.829 = 0.829 QALY). QALY value of 2^nd^ generation DES was used as baseline and change was calculated in QALYs value for newer generation BP-DES.


QALYgained=0.829–0.794=0.035QALYs

**Fig 2:**
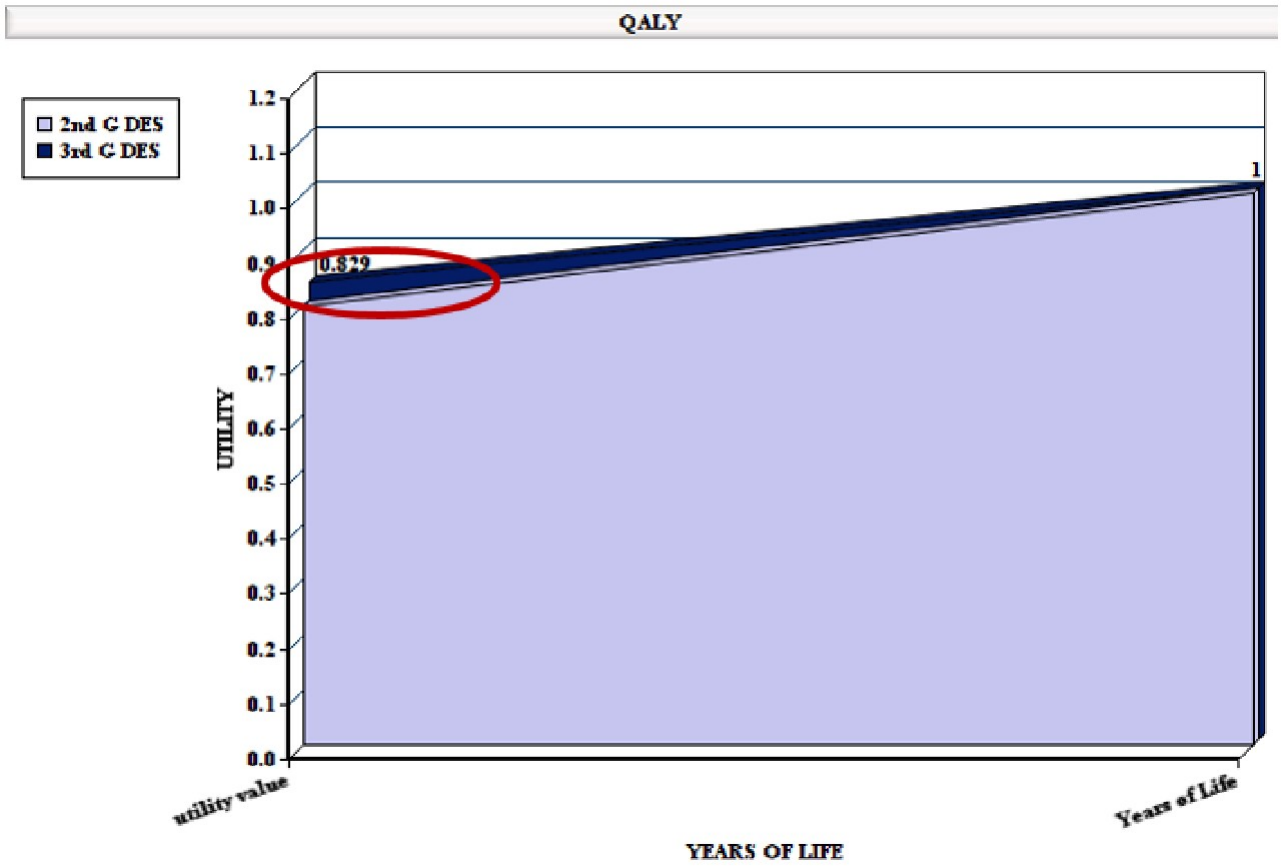
QALY gained by 3rd G DES (QALYs gained is marked by red circle).

Hence by the use of 3^rd^ G drug eluting stents 0.035 QALY were gained as compared to the use of 2^nd^ G DES (Fig 2).

### EQ-5D-5L visual analogue scale values

Values of EQ-VAS were obtained on a scale from 0–100, where patients marked their health state, thus it gives QoL from the patient’s perspective. EQ-VAS scores were summarized as mean and standard deviation. Overall mean value of visual analogue scale was 79.68 ± 8.84. (Min. value = 60 and max. value = 95). The distribution of VAS values with demographic, angiographic variables and risk factors are presented in Table 4. The mean value of VAS for 3^rd^ generation BioMatrix series BP-DES was higher 81.48 ± 8.29 than 2^nd^ G Xience series DP-DES; 77.81 ± 9.01. The mean VAS value was high in males (80.06 ± 8.60), age group 39–57 years (81.17 ± 8.59) and rural area residents (80.41 ± 8.28). VAS value in hypertensive (75.21 ± 8.58), diabetic patients (74.47 ± 8.70), smokers (75.69 ± 8.26) and hyperlipidemia patients (76.55 ± 8.62) was lower than patients having no risk factor. VAS mean value in patients having family history of CAD was low i.e., 77.61 ± 8.713, then without family history.

**Table 4:** Distribution of VAS values among independent variables.

Sr. No	Variable	Categories	Frequency	Mean VAS value & SD
**1**	**Gender**	Male	291	80.06 (8.60)
		Female	43	77.09 (10.01)
**2**	**Age**	19–38	7	80.57 (9.14)
		39–57	143	81.17 (8.59)
		58–75	175	78.46 (8.86)
		> 75	9	79.11 (9.94)
**3**	**Residence**	Urban	234	79.37 (9.07)
		Rural	100	80.41 (8.28)
**4**	**Occupation**	Unemployed	160	79.79 (8.75)
		Employed	174	79.58 (8.9)
**5**	**DM**	Yes	68	74.47 (8.70)
		No	266	81.01 (8.38)
**6**	**HTN**	Yes	110	75.21 (8.58)
		No	224	81.88 (8.12)
**7**	**Hyperlipidemia**	Yes	66	76.55 (8.62)
		No	268	80.45 (8.74)
**8**	**Current Smoking**	Yes	59	75.69 (8.26)
		No	275	80.53 (8.74)
**9**	**Family History**	Yes	95	77.61 (8.713)
		No	239	80.5 (8.77)
**10**	**Type of MI**	ANT MI	226	79.85 (9.02)
		INF MI	108	79.32 (8.47)
**11**	**CAD Diagnosis**	SVCAD	129	81.48 (8.25)
		DVCAD	108	79.35 (9.63)
		TVCAD	96	77.56 (8.25)
**12**	**DES Type**	2^nd^ G	164	77.81 (9.01)
		3^rd^ G	170	81.48 (8.29)

A multiple regression/multiple linear regression analysis was run to predict the VAS value from age, gender, DM, HTN, hyperlipidemia, family history, smoking status, type of MI, DES type and CAD diagnosis. These variables statistically significantly predicted the VAS, F (10, 322) = 14.907, p < 0.0005, R^2^ = 0.316, Adjusted R = 0.295. Except 2 variables all others have a statistically significant relation with response variable (VAS) P < 0.05. A significant association was found between VAS and types of DES [P<0.05; CI (B) -5.367 - -2.119]. Seven other variables were also found to have a significant association with VAS values, these include age, diabetes mellitus, hypertension, current smokers, family history of CAD, type of MI and CAD diagnosis (p< 0.05). Gender and hyperlipidemia were not associated significantly with visual analogue scale (Table 5).

**Table 5:** Association of EQ-VAS values with independent variables.

Sr. No.	Variable	Unstandardized Co-efficient (B)	Std. Error	p-value	95% CI range
**1**	**Age**	-1.608	0.709	0.024	-3.002 - -0.214
**2**	**Gender**	-1.860	1.287	0.149	-4.392–0.671
**3**	**DM**	4.952	1.060	0.000	2.866–7.038
**4**	**Hypertension**	5.304	0.926	0.000	2.560–6.205
**5**	**Current Smokers**	5.304	1.100	0.000	3.140–7.467
**6**	**Hyperlipidemia**	2.054	1.062	0.054	-0.035–4.143
**7**	**Family History**	2.045	0.934	0.029	0.209–3.882
**8**	**DES Type**	-3.743	0.826	0.000	-5.367 - -2.119
**9**	**Type of MI**	-0.191	0.879	0.828	-1.921–1.539
**10**	**CAD Diagnosis**	-1.155	0.510	0.024	-2.157 - -0.152

## 4. Discussion

Newer generation biodegradable polymer stents are being used as a standard of care in PCI. Many studies have been conducted for comparison of BP-DES and DP-DES, however, to our knowledge this is the first comparative study of 2^nd^ generation DP-Xience series (Xience Prime & Xience Xpedition) stents with the 3^rd^ generation biodegradable polymer Biomatrix (Biomatrix Alpha & Biomatrix Neoflex) stents in the Pakistani population to assess the quality of life in CAD patients after primary percutaneous coronary intervention. PPCI is the preferred treatment option in STEMI, the aim is to open the blockage of a vessel as soon as possible [25]. The results of our study showed that the QoL values of 3^rd^ G stent were higher than 2^nd^ G (utility values: P<0.05 and VAS values: P< 0.05). Most of the patients undergoing PPCI at study center were males (85.6%), same was reported in many other studies on PCI, like studies carried out at lady reading hospital Peshawar (males; 76.2%) [26], Kulsum International hospital, Islamabad (males: 86%) [25] and a multicenter study in India (males; 81.8%) [17].

CAD is the major cause of mortality and morbidity in world, according to a report about 80% of the total cardiovascular related deaths occur in low- and middle-income countries [20]. Poor QoL is an important outcome of chronic disease patients, like CAD. Studies related to PCI mostly focus on treatment effectiveness, thus there are very few studies available on QoL after PCI [27]. A study comparing HRQOL of PCI vs CABG concluded that the QoL in patients of PCI is more than CABG at 6 months, however, after 2 years of procedure QoL was same in both groups [20]. We have used EQ-5D-5L questionnaire in our study which has two portions, the results are thus obtained in the form of utility values for the first portion and VAS values for the second portion. Utilities give us the individual’s preference for a particular health outcome. Moreover, utilities are also employed to calculate QALYs, which are used for economic evaluation [15].

The mean value of utility obtained in our study was 0.812± 0.11; 2^nd^ G DP-DES (Xience) was 0.794 ± 0.11 and 0.829 ± 0.11 for BP-DES (Biomatrix). Mean VAS values for DP stents was 77.81 ± 9.01 and for BP stents was 81.84 ± 8.29 (mean VAS score 79.68 ± 8.84). Thus BP-DES (Biomatrix neoflex/Biomatrix Alpha) were found to improve the patient’s QoL as compared to DP-DES (xience prime/Xience Alpha), significant association was found in statistical analysis [utility values (P < 0.05)) and VAS values (P < 0.05)]. HRQOL comparison of various treatment options for CAD (PCI, CABG and medical therapy) after ACS was assessed in a study using EQ-5D-5L and VAS, results concluded that PCI has comparable HRQOL with CABG and better as compared to medical therapy [28].

QoL (patient reported outcomes) was assessed in patients of primary PCI after three months of procedure using Seattle angina questionnaire and EQ-5D-3L. EQ-5D-3L index score at baseline vs. at 3 months was 0.79 vs 0.82, and was not much different at follow-up [29]. There are very few studies comparing QoL of cardiac stents after PCI and PPCI, and no published study has been found comparing utility values or QALYs of Biomatrix and Xience DES.

Many clinical, demographic and psychological variables are found to be associated with HRQOL [30]. Gender and age are well known predictors of QoL [20]. Female patients with CAD have more age than male counterparts and therefore experience comparatively poorer HRQOL [31]. Our study has found a significant association between utility value and gender (P = 0.042) but not with VAS value, however, the mean VAS value was more in males than females. In age groups, 39–57 years was having the highest value of utility score and VAS, significant association was found b/w age and utility values and b/w age and VAS values. A study conducted at Korea found significant association of dyslipidemia and QoL, and concluded that dyslipidemia is associated with poor HRQOL [11]. HRQOL in old age people ≥ 70 years and below 70 was assessed in a study using Medical Outcomes Short form-12 (SF-12) and Seattle Angina Questionnaire (SAQ). Age, gender, number of stents and hospital stay duration were found as predictors of QoL, study concluded that PPCI improves HRQOL as measured 4–6 weeks after the procedure [18]. Other studies found a significant association of gender, age, DM, body mass index, previous MI history, depression, smoking and treatment type with HRQOL in CAD patients [30, 32].

MI, vascular diseases and HTN have been found as predictors of HRQOL in few studies [20]. Our study has found a significant association between utility value, VAS and hypertension, hyperlipidemia, diabetes mellitus and current smoking history. Mean utility value and VAS score values were high in non- hypertensive patients (0.844 ± 0.10; 81.88 ± 8.12), non-DM (0.829 ± 0.11; 81.01 ± 8.38), patients having no hyperlipidemia (0.823 ± 0.11; 80.45 ± 8.74) and non-smokers (0.825 ± 0.11; 80.53 ± 8.74). Association of smoking and HRQOL after PCI was assessed in a study that grouped patients as non-smokers, ex-smokers, quitters and current smokers. Persistent/current smokers were having worse QoL as compared to other groups, and a significant association was found between QoL and smoking at one year after PCI, QoL was assessed by using EQ-5D questionnaire and Seattle angina questionnaire [12].

QALY is the measurement of improvement in quality and quantity of life. QALY is obtained by multiplying quality (utility values) and quantity (years of life). QALY is a valid index for clinical and economic evaluations of PCI. In the current study, value of QALY for DP-DES was 0.794 and for BP-DES it was 0.829, there was a gain of 0.035 QALYs by use of BP-DES. Thus, the use of BP stent was associated with an increase in QALYs of patients. In an economic analysis study comparing 2^nd^ and 3^rd^ generation DES, utility and QALYs values obtained at one year follow-up were 0.7781 and 0.7595 for BP-DES and DP-DES respectively, utility was obtained using EQ-5D-5L questionnaire [33]. Comparative studies on QALY are not available. QALY values are used in economic evaluation (cost utility analysis).

### Limitations

This study has been conducted at a single center so the sample may not be representative of the whole population undergoing PPCI. Stent selection bias may exist, and it was on cardiologist discretion and the data was retrieved from the record. The severity of the disease was not accounted in the study. For establishing convincing evidence, longer follow-up studies and randomized controlled trials are required.

## 5. Conclusion

Newer generation drug eluting stents have been presented as a novel solution to the problems of 2^nd^ generation stents. HRQOL is a leading sport in decision making of therapeutic interventions. Healthcare decision makers need rapid access to information, and evidence that assist them to draw conclusion are often not available. Utility values and VAS scores were higher for BP stents indicating better outcomes of 3^rd^ G DES. There was a gain of 0.035 QALY by the use of 3^rd^ G DES. Utility values and QALY are important outcome measures for the assessment of healthcare interventions as they are employed in economic evaluation and assist healthcare decision makers.

### Future perspectives

This study’s results will provide valuable information to the decision makers about the use of newer generation DES and effective management of limited healthcare resources to obtain maximum benefits. The results of this study can be implemented in pharmaco-economic analysis and can assist policymakers in the allocation of scarce healthcare resources for expensive therapies and in the management of chronic diseases like CAD.

## Supporting information

S1 File Highlights. (DOCX)

## Abbreviations

BES: Biolimus eluting stent
BMS: Bare metal stents
BP: Biodegradable polymer
CABG: Coronary artery bypass graft
CAD: Coronary artery disease
DES: Drug eluting stents
DM: Diabetes Mellitus
DP: Durable polymer
EES: Everolimus eluting stent
EQ-5D-5L: EuroQol -5-dimensions-5-levels
HRQOL: Health related quality of life
HTN: Hypertension
PCI: Percutaneous coronary intervention
PPCI: Primary percutaneous intervention
QALY: Quality adjusted life year
QoL: Quality of life
STEMI: ST elevated myocardial infarction
