# Supplementary material for: Assessment of utility values and QALYs after primary PCI with DP-Xience and BP-Biomatrix stents
Source: PLoS One. 2021 Jun 17;16(6):e0253290. doi: 10.1371/journal.pone.0253290 (PMC8211164; doi:10.1371/journal.pone.0253290)
Supplement: S1 File — (DOCX) [file pone.0253290.s001.docx]

**Highlights**

- Determination of QOL of various interventions is very important in the establishment of a comprehensive care plan.
- Biomatrix 3^rd^ G drug eluting stents have better QOL than Xience 2^nd^ G stents after PPCI.
- Smoking, family history of CAD, HTN and DM are significant predictors of QOL.
- QALY is employed in economic evaluation to guide clinicians and policymakers in the allocation of healthcare resources.
